# Antioxidant and Anti-Inflammatory Activity of Combined Phycocyanin and Palmitoylethanolamide in Human Lung and Prostate Epithelial Cells

**DOI:** 10.3390/antiox11020201

**Published:** 2022-01-21

**Authors:** Loredana Bergandi, Giulia Apprato, Francesca Silvagno

**Affiliations:** Department of Oncology, University of Torino, Via Santena 5 bis, 10126 Torino, Italy; loredana.bergandi@unito.it (L.B.); giulia.apprato@gmail.com (G.A.)

**Keywords:** phycocyanin, palmitoylethanolamide, ROS, inflammation, cytokines, glutathione, mitochondrial respiratory complexes, human lung epithelial cells, human prostate epithelial cells

## Abstract

Inflammation involving the innate and adaptive immune systems is a normal response to infection; however, when allowed to continue unchecked, inflammation may result in several pathologies. Natural molecules with antioxidant properties can target the key players of inflammation and exert beneficial health effects. In this study, human normal bronchial (Beas-2B) and prostate (HPrEpiC) epithelial cell lines were exposed to infectious stimulation and treated with phycocyanin (PC) and palmitoylethanolamide (PEA), with the aim of demonstrating the enhanced antioxidant and anti-inflammatory properties of the combination. The cotreatment protected from cytotoxicity and greatly abated both the production of radical oxygen species (ROS) and the transcription of several inflammatory cytokines. Oxidative stress and inflammation were curtailed by affecting three main pathways: (1) inhibition of cyclooxygenase-2 enzyme and consequent decrease of signaling generating ROS; (2) increased synthesis of glutathione and therefore strengthening of the natural antioxidant defenses of the cells; (3) decreased infection-driven mitochondrial respiratory burst which generates oxidative stress. Based on the mounting interest in using nutraceuticals as adjuvants in the clinical practice, the present study unveils new mechanisms of action and enhanced efficacy of PC and PEA, supporting the possible exploitation of this combination in human disorders.

## 1. Introduction

The goal of the inflammatory process is the full recovery to a state of good health and the return to conditions of homeostasis [1]. The inflammatory phenomenon, resulting from the clearance of the infectious agent and the repair of the affected tissues, represents a defensive strategy guided by biological molecules (neuropeptides, hormones, cytokines, growth factors). However, its failure can induce the progression of the pathology, the onset of functional and structural damage, and thus of a chronic inflammatory process, often with a fatal outcome [1]. To recruit additional immune cells to the site of infection, inflammation triggers the secretion of various cytokines and chemokines by immune cells [2]. When the homeostatic capacity is insufficient, mechanisms are incapable of resolving the insult; when it is excessive, a stress inflammatory response is engaged [3].

There are many intracellular signaling pathways that respond to inflammation. Among them, the activation of the nuclear transcription factor NF-κB, and the production of radical oxygen species (ROS) are the main mediators on which converge several inflammatory stimuli. NF-κB and ROS signaling are strictly linked and stimulate each other. Several signals such as inflammatory cytokines, LPS (bacterial lipopolysaccharide), viral particles and other mediators that activate TLRs (Toll-Like Receptors), known as pathogen-associated molecular patterns (PAMPS), converge on the TLR/NF-κB axis. In fact, the microbial and viral components, LPS and Poly(I:C) (TLR4 and TLR3 agonists, respectively), trigger the activation of two downstream signaling pathways of TLRs. The MyD88- and TRIF-dependent pathways (Myeloid differentiation factor 88 and TIR domain-containing adapter inducing IFN-beta) lead to the activation of NF-κB and IRF3 and of the downstream target cyclooxygenase-2 (COX-2) [4,5,6]. Indeed, it is known that NF-κB induces the expression and activity of the enzyme COX-2 [7], which produces various derivatives of arachidonic acid, such as prostaglandins (PG). Among them, PGE2 (prostaglandin E2) possesses proinflammatory activity, resulting in the secretion of inflammatory cytokines that mediate the activation of NF-κB with amplification of the inflammatory response.

A vicious circle between ROS and COX-derived products has been described by few studies. On one hand, both the activity and the expression of COX isoforms are affected by ROS produced from different sources because oxidative stress mediates COX-2 expression induced by LPS, IL-1β (Interleukin-1β) and tumour necrosis factor-α (TNF-α) in different cell types [8]. On the other hand, COX metabolites from arachidonic acid may activate ROS production via NADPH oxidase stimulation [9].

ROS are originated not only by environmental or pharmacological exposure, but are also produced in excess by a mitochondrial metabolic derangement. Oxidative stress (OS) and tissue injury are caused by enhanced ROS generation triggered by immune cells at the site of inflammation [10]. Indeed, OS occurs due to the imbalance between the production of ROS and the availability of antioxidants or radical scavengers [11]. Glutathione represents the main antioxidant defense of the tissues. The reciprocal regulation of glutathione and COX-2 has been described previously. In fact, it has been reported that the mutual regulation between the glutathione-dependent glutathione peroxidase (GPx) and COX-2 activity and expression of GPx [12,13] by local removal of hydroperoxides silences COX-2 activity and suppresses COX-2 expression. When the glutathione levels are low, the excess of ROS production can either oxidize biomolecules or can structurally modify proteins and genes to trigger signaling cascades through the activation of transcription factors and pro-inflammatory genes. These events can lead to the onset and progression of inflammatory diseases such as aging, neurodegenerative disorders, and cancer [14]. Different pharmacological approaches are continuously developed in the effort of decreasing inflammation by targeting either COX-2 activity or ROS production.

In addition to synthetic drugs, spirulina and its bioactive phycocyanin extract (PC) have been proposed as selective inhibitors of COX-2. PC participates in the algal photosynthesis process due to its ability to help capture light energy efficiently. Its chemical structure makes this molecule a free radical scavenger, and its antioxidant and antitumor activity has been described [15]. The antioxidant, immunomodulatory and anti-inflammatory properties of PC have been demonstrated both in animal models and in humans [15,16]. PC scavenges both reactive oxygen and nitrogen species [17], preventing oxidative damage through the inhibition of COX-2 that may explain, at least in part, its beneficial effects [18]. A recent study on mice showed that in pulmonary fibrosis PC inhibits the TLR2/NF-κB axis and reduces the production of inflammatory cytokines and OS [19]. Moreover, paraquat-induced and LPS-induced acute lung injury was prevented by PC by avoiding oxidative damage and inhibiting NF-κB-mediated cytotoxicity [20,21] in a rat model.

Another molecule proposed by recent studies as an anti-inflammatory substance is palmitoylethanolamide (PEA). PEA exerts anti-inflammatory, analgesic and neuroprotective actions [22]. PEA activates the PPARα (peroxisome proliferator-activated receptor α), which decreases inflammation by acting on the TLR/NF-κB axis, reducing the activity of NF-κB, inhibiting the expression of inflammatory cytokines such as TNF-α and limiting the recruitment of immune cells. A recent study reports that PEA in combination with paracetamol decreases inflammation mediated by NF-κB and, consequently, reduces the production of inflammatory prostaglandins caused by the activity of COX-2 [23]. The effect of PEA on COX-2 was, therefore, described as an indirect effect, mediated by NF-κB. Importantly, evidence suggests that during inflammation PEA metabolism is disturbed, and that a decrease in PEA levels contributes to the inflammatory response [22].

Based on this knowledge, a combined treatment with PC and PEA could reveal novel antioxidant and anti-inflammatory effects, superior to the properties of the single molecules. To date, the potentiation of this combination of molecules has not been tested. The aim of this work was to assess the effects of the combination in the presence of an inflammatory stimulation, and to investigate the possible underlying mechanisms. 

## 2. Materials and Methods

### 2.1. Cell Culture and Treatments

Human normal bronchial (Beas-2B) and prostate (HPrEpiC) epithelial cell lines were purchased from the American Type Culture Collection (ATCC, Manassas, VA, USA) and from CliniSciences (Guidonia Montecelio, Rome, Italy), respectively. Beas-2B cells were cultured in RPMI medium supplemented with 10% fetal bovine serum and 1% antibiotics (penicillin-streptomycin) at 37 °C in a humidified 5% CO_2_ atmosphere. HPrEpiC cells were cultured in prostate epithelial cell medium supplemented with 10% fetal bovine serum and 1% penicillin-streptomycin at 37 °C in a humidified 5% CO_2_ atmosphere in Poly-l-Lysine coated dishes, following the vendors protocol. Then, Beas-2B and HPrEpiC cells were treated with 1 µM phycocyanin (PC) and 1 µM palmitoylethanolamide (PEA) for 24 h in a single treatment or cotreatment in the absence or presence of 20 µg/mL lipopolysaccharide (LPS, which mimics the bacterial stimulus) or of 10 µg/mL Polyinosinic:polycytidylic acid (Poly(I:C), which mimics the viral stimulus) for Beas-2B and of 40 µg/mL for HPrEpiC cell culture. PC and PEA were obtained from Kura srl (Torino, Italy). Unless otherwise specified, plasticware was from Falcon (Becton Dickinson, Franklin Lakes, NJ, USA) and reagents were purchased from Merck (Milan, Italy). 

### 2.2. Human Recombinant COX-2 Inhibitor Screening Assay

COX-2 (also called prostaglandin H synthase 2, PGH2) IC50 was determined by the human COX-2 inhibitor screening assay according to the manufacturer’s protocol (Cayman Chemical, Ann Arbor, MI, USA). The human COX-2 inhibitory assay directly measures, via enzyme immunoassay (EIA), the prostaglandin F2α (PGF2α) that is produced by SnCl_2_ reduction of COX-2-derived PGH2, which is produced in the in vitro COX reaction. 

### 2.3. Cytokines Quantification

After treatments, the concentrations of PGE2, IL-6 and IL-8 were determined in culture supernatants by enzyme-linked immunosorbent assay, according to the manufacturer’s protocol (Cayman Chemical, Ann Arbor, MI, USA and R&D Systems, Inc., Minneapolis, MN, USA). Analyte concentrations in supernatants were expressed as pg/mg cellular proteins for PGE2, whereas the levels of IL-6 and IL-8 were expressed as percentage of control. 

### 2.4. Measurement of Intracellular ROS Production

After treatments, the cells were harvested and loaded for 15 min with 10 μM 2′,7′-dichlorodihydrofluorescein diacetate (DCFH-DA, Merck, Milan, Italy), as previously described [24,25]. The fluorescence values were expressed as relative fluorescence units (RFUs) and were normalized to the protein content. 

### 2.5. Toxicity Assay (LDH Release)

After treatments, cell damage was evaluated by measuring the release of lactate dehydrogenase (LDH) in the growth medium as previously described [25,26]. Protein content was quantified by the BCA protein assay (Merck, Milan, Italy) and the enzymatic activity was expressed as µmol NADH oxidized/min/mg cell protein. 

### 2.6. Measurement of Total Glutathione (GSH)

Total cellular GSH contents were determined using a commercial kit according to the manufacturer’s protocol (Cayman Chemical, Ann Arbor, MI, USA) that utilizes a carefully optimized enzymatic recycling method, using glutathione reductase for the quantification of GSH and an alternative protocol to measure only GSSG. The glutathione content was calculated as nmol/mg protein based on cellular protein concentrations and expressed relative to control. 

### 2.7. Real-Time Polymerase Chain Reaction (qRT-C-PCR)

Total RNA was extracted with TRIzol^®^ (Invitrogen, Thermo Fisher Scientific, Waltham, MA, USA) after treatments. One μg of total RNA was reversely transcribed into cDNA using an iScript cDNA Synthesis Kit (Bio-Rad Laboratories AG, Cressier FR, Switzerland) according to the manufacturer’s instructions. The RT-PCR primers were designed with NCBI/Primer-BLAST and synthesized by Merck (Milan, Italy). Quantitative PCR was carried out in a final volume of 20 μL using the IQ™ SYBR Green Supermix (Bio-Rad Laboratories AG, Cressier FR, Switzerland) with specific primers for the quantitation of the following human genes: IL-8 [27], IL-6 [28], TNF-α [29], cytochrome *c* oxidase subunit 2 (COXII) [30], cytochrome *c* oxidase subunit 4 (COXIV) [30], ATP synthase subunit beta (ATP5B) [30], mitochondrial ATP synthase F0 subunit 6 (MT-ATP6) [30], glutamate-cysteine ligase catalytic subunit (GCLC) [31] and beta 2-microglobulin (β2M) [25]. Gene accession number of amplified human genes are reported in supplementary Table S1. PCR amplification was conducted with 1 cycle of denaturation at 94 °C for 32 min, 45 cycles of amplification including denaturation at 94 °C for 30 s and annealing/extension at 60 °C for 30 s. Data were analysed using the 2^−^^ΔΔCT^ method as transcripts exhibited high linearity amplification plots (*r* > 0.97), and β2M was used as the reference gene to normalize the cDNA in different samples. Nonspecific amplifications were not detected, and the specificity of PCRs was confirmed by melt curve analysis.

### 2.8. Statistical Analysis

Statistical analysis of data was performed using ANOVA test with Tukey’s post-hoc correction. *p* values < 0.05 were considered significant. All data ae expressed as mean ± S.E.M. of three independent experiments.

## 3. Results

### 3.1. PC and PEA Inhibit COX-2 Activity

As PC exerts its anti-inflammatory activity through the inhibition of COX-2 enzymatic activity, we sought to confirm this property as well as explore the possible synergy with PEA, another anti-inflammatory compound. In fact, the direct influence of PEA on COX-2 driven inflammation has not yet been investigated. 

To verify the inhibitory activity of PC and PEA drugs, alone or in combination, a human recombinant COX-2 inhibitor screening test was initially performed in a cell-free enzyme assay. The in vitro analysis of COX-2 activity revealed the inhibition exerted by both molecules. We calculated the IC50 for PC as 0.36 µM, similar to values found by other studies in vitro [18]. Interestingly, a novel property of PEA as a direct COX-2 inhibitor was unveiled, since the molecule showed a IC50 value of 0.57 µM. Most intriguingly, the inhibitory effect was enhanced by the combination of the two compounds, leading to a IC50 value of 0.038 µM, demonstrating a potentiation of ten times compared to PC alone. Dose-dependent effects of PC and PEA were demonstrated by analysis of the inhibition exerted on COX-2 enzymatic activity, which showed the potentiated inhibitory effect caused by the combination compared to single molecules (Supplementary Figure S1). Considering that in biological systems drug absorption and pharmacokinetic parameters, as well as the choice of cell model and treatment [18], could vary the efficacy of the two compounds, all experiments carried out in cells used the higher amounts of the two molecules, namely 1 µM PC and 1 µM PEA. This concentration was selected in previous studies in vitro (reviewed in [17]), and was chosen as the most appropriate in our effort of establishing the effects of the combination in cells. 

**Figure 1:**
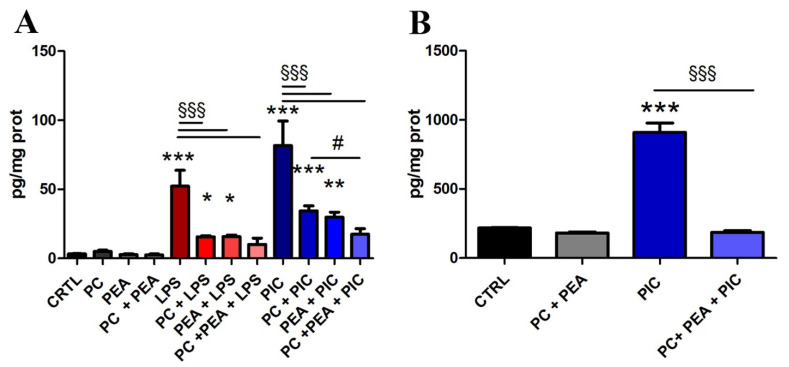
PC and PEA inhibit COX-2 activity. Cells were treated for 24 h either with 20 µg/mL bacterial antigen lipopolysaccharide (LPS) or with 10 µg/mL Poly(I:C) (PIC). Moreover, together with the inflammatory stimulation the cells were treated for 24 h with 1 µM PC and 1 µM PEA, alone or in combination, and the level of PGE2 was measured in culture medium of Beas-2B (**A**) and HPrEpiC (**B**) cells. Control cells (CTRL) were not treated. Results are means ± SEM. * *p* < 0.05, ** *p* < 0.01 and *** *p* < 0.001 vs. ctrl; ^§§§^ *p* < 0.001; # *p* < 0.05. The latter statistical significance demonstrates potentiation of the effect when the combination is used vs. single molecules.

To confirm the novel observations acquired in the in vitro assay, we decided to test the effects of the two molecules, alone or in combination, on two models of epithelial cells (lung and prostate) extracted from tissues subjected to inflammatory damage and oxidative stress. In previous research, PC activity was investigated in lung tissue of animal models [19,20,21] and in human lung cancer cell lines [32]. PEA anti-inflammatory properties were studied in lung [33] and in prostatic tissues [34] of animal models, but the combination was never proposed. Beas-2B is a human pulmonary epithelial cell line frequently used in studies on inflammation. The inhibitory effect on COX-2 activity was tested in these cells as secretion of PGE2, a product synthesized by the enzyme upon stimulation with inflammatory molecules. For this purpose, Beas-2B cells were treated either with LPS or with Poly(I:C). Poly(I:C) is structurally similar to dsRNA, present in some viruses, and it is commonly used to model the actions of extracellular dsRNA [35]. The measurement of PGE2 in the culture medium confirmed its induced synthesis after inflammatory stimuli and demonstrated the inhibition of COX-2 activity after single treatments with PC and PEA, as shown in Figure 1A. Most importantly, we found confirmed the potentiating effect of the combination of PC and PEA, because the single treatments only partially abated the LPS-induced secretion of PGE2, whereas the levels of PGE2 after cotreatment with LPS, PC and PEA were not significantly different from untreated control cells. The potentiation was more evident upon inflammatory stimulation with Poly(I:C), as we could observe a significative difference between PC + PIC and PC + PEA + PIC treatments (Figure 1A). The same inhibitory effect of COX-2 was observed in the model of human prostate epithelial cells, in which PC and PEA abated the synthesis of PGE2 in response to Poly(I:C) (Figure 1B). In this assay, and in further experimental settings, in prostate epithelial cells we tested only the most potent inflammatory stimulation with Poly(I:C), which was more consistent in terms of potentiation of the effect with the combination PC + PEA.

### 3.2. PC and PEA Protect Cells from Oxidative Stress and Damage

**Figure 2:**
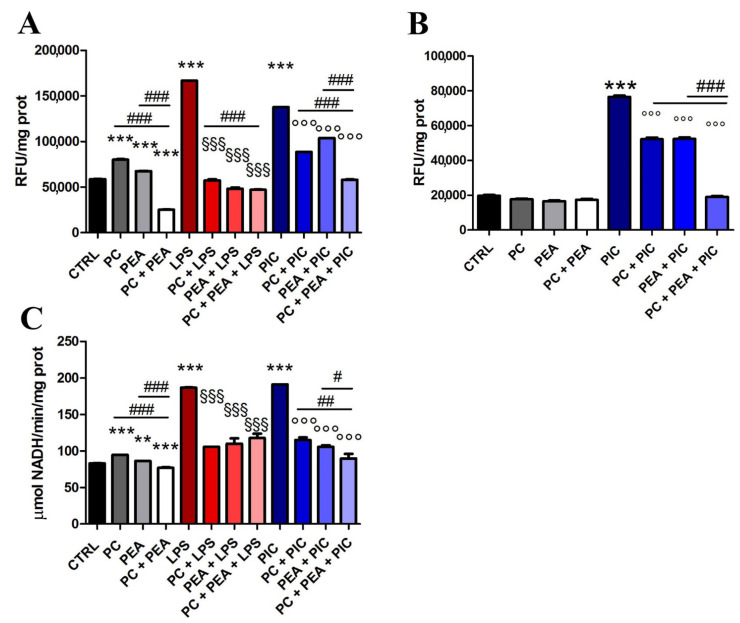
PC and PEA protect cells from oxidative stress and damage. Cells were treated for 24 h either with 20 µg/mL bacterial antigen lipopolysaccharide (LPS) or with 10 µg/mL Poly(I:C) (PIC). Cells were also treated for 24 h with 1 µM PC and 1 µM PEA, alone or in combination. The intracellular level of ROS was measured both in Beas-2B (**A**) and HPrEpiC (**B**) cells. The values expressed as RFU (Relative Fluorescence Unit) were normalized to protein content. (**C**) The release of LDH was measured in Beas-2B in the culture medium and expressed as enzymatic activity in relation to protein content. Control cells (CTRL) were not treated. Results are means ± SEM. ** *p* < 0.01 and *** *p* < 0.001 vs. ctrl; ^§§§^ *p* < 0.001 vs. LPS; ^°°°^ *p* < 0.001 vs. PIC. # *p* < 0.05, ## *p* < 0.01 and ### *p* < 0.001. The latter statistical significance demonstrates the potentiation of the effect when the combination is used vs. single molecules.

To verify if the activation of COX-2 induced ROS production, resulting in cell toxicity, and to assess the impact of PC and PEA on this pathway, we first tested whether the two compounds affected oxidative stress. We discovered that PC and PEA, alone or in combination, protected against ROS production and cytotoxicity induced by the inflammatory stimuli. Indeed, LPS- and Poly(I:C)-induced ROS production was inhibited by single molecules and even more by the cotreatment, both in human normal bronchial (Figure 2A) and prostate epithelial cell lines (Figure 2B). Similarly, when we tested cytotoxicity by the LDH release assay, we found that PC and PEA, alone and in combination, decreased the cellular damage measured as enzymatic activity abnormally detected in the culture medium (Figure 2C).

### 3.3. PC and PEA Enhance the Antioxidant Defenses of the Cells

The previous results suggested that PC and PEA protected from oxidative stress triggered by the production of ROS. As the well-known antioxidant sulfhydryl tripeptide glutathione (GSH) is important for the maintenance of the cellular redox state and the prevention of oxidative injury [36], we sought to investigate whether PC and PEA could modulate GSH levels. 

**Figure 3:**
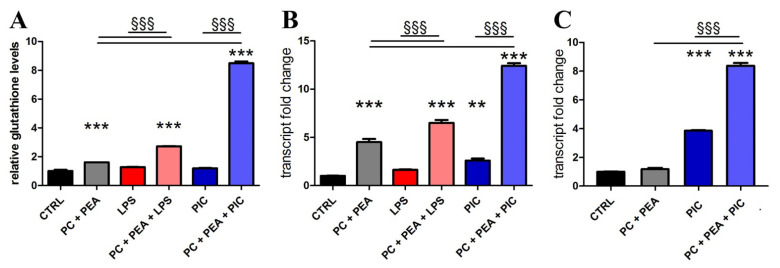
PC and PEA potentiate the antioxidant defenses of the cells. Cells were treated for 24 h with 20 µg/mL bacterial antigen lipopolysaccharide (LPS) or with 10 µg/mL Poly(I:C) (PIC), 1 µM PC and 1 µM PEA, alone or in combination. The amount of total GSH was measured in Beas-2B (**A**) and the mRNA expression of glutamate-cysteine ligase catalytic subunit (GCLC) was detected by qRT-PCR both in Beas-2B (**B**) and HPrEpiC (**C**) cells. Control cells (CTRL) were not treated. Results are means ± SEM. ** *p* < 0.01 and *** *p* < 0.001 vs. ctrl; ^§§§^ *p* < 0.001. The latter statistical significance demonstrates the potentiation of the effect when the combination PC + PEA is used in the presence of inflammatory stimuli vs. the inflammatory stimuli alone or PC + PEA only.

The intracellular glutathione assay demonstrated that the treatment with PC and PEA was able to enhance GSH levels, and such increase was potentiated by the presence of an inflammatory stimulus (LPS or Poly(I:C), as shown in Figure 3A. Indeed, in Beas-2B cells, the incubation with PC, PEA and the inflammatory stimulus seemed to have an addictive effect, possibly mediated by the induction of the endogenous synthesis of glutathione. To verify this assumption, we measured the transcription of a key enzyme in GSH biosynthesis, namely the catalytic subunit of the glutamate-cysteine ligase (GCLC) enzyme [37]. We discovered that both in Beas-2B (Figure 3B) and prostate HPrEpiC (Figure 3C) the two molecules acted on gene transcription of the enzyme that drives GSH production.

### 3.4. The Anti-Inflammatory Activity of PC and PEA Is Enhanced by the Combined Treatment

It is well known that LPS/Poly(I:C) induces cell injury mainly through inflammation [38]. Generally, inflammation is caused by the release of pro-inflammatory cytokines, including IL-6, IL-8 and IL-1β, and TNF-α from natural killer cells, T lymphocytes and macrophages [38]. 

**Figure 4:**
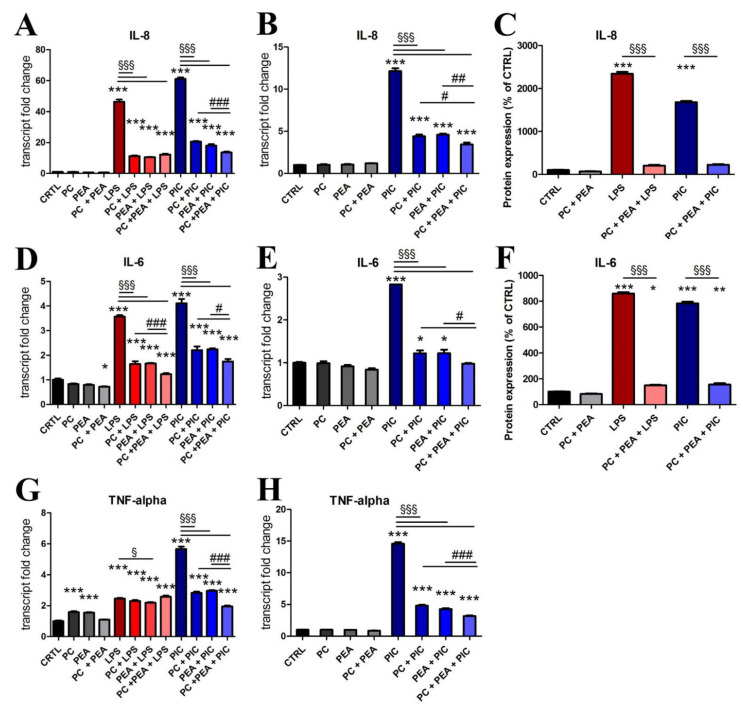
PC and PEA potentiate the anti-inflammatory activity of the cells. Cells were treated for 24 h with 20 µg/mL bacterial antigen lipopolysaccharide (LPS) or with 10 µg/mL Poly(I:C) (PIC), 1 µM PC and 1 µM PEA, alone or in combination. Control cells (CTRL) were not treated. The transcript levels IL-8, IL-6 and TNF-α were detected by qRT-PCR both in Beas-2B (**A**,**D**,**G**) and HPrEpiC (**B**,**E**,**H**) cells. Secreted cytokine expression was measured in culture medium of Beas-2B cells (**C**,**F**). Results are means ± SEM. * *p* < 0.05, ** *p* < 0.01 and *** *p* < 0.001 vs. ctrl; ^§^ *p* < 0.05 and ^§§§^ *p* < 0.001; # *p* < 0.05, ## *p* < 0.01, and ### *p* < 0.001. The latter statistical significance demonstrates the potentiation of the effect when the combination is used vs. single molecules.

To verify the efficiency of PC and PEA in immunomodulation responsive to LPS/Poly(I:C), we used qRT-PCR to determine IL-8, IL-6 and TNF-α mRNA expression. We found that cotreatment with PC and PEA enhanced the anti-inflammatory properties of the individual molecules, abating the LPS/Poly(I:C)-induced upregulation of IL-8, Il-6 and TNF-α both in Beas-2B (Figure 4A,D,G) and prostate HPrEpiC cells (Figure 4B,E,H). The potentiated effect of the combination compared to single molecules was particularly evident when cells were stimulated with Poly(I:C). Moreover, we verified the anti-inflammatory effect of PC and PEA by protein expression analysis. The measure of secreted cytokines confirmed that treatment with PC + PEA was able to drastically reduce the stimulated secretion of cytokines (Figure 4C,F).

### 3.5. PC and PEA Decrease the Respiratory Burst Triggered by the Inflammatory Stimulus 

**Figure 5:**
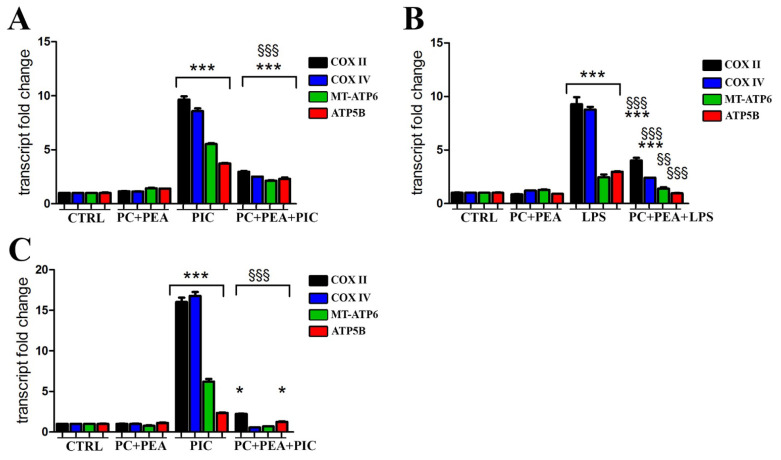
PC and PEA synergically decrease the respiratory burst triggered by the inflammatory stimulus. Cells were treated for 24 h either with 20 µg/mL bacterial antigen lipopolysaccharide (LPS) or with 10 µg/mL Poly(I:C) (PIC), 1 µM PC and 1 µM PEA, alone or in combination. The transcript levels of cytochrome C oxidase subunit 2 (COX II) and subunit 4 (COXIV), and the transcription of two subunits of ATP synthase (MT-ATP6 and ATP5B) were measured by real-time PCR both in Beas-2B (**A**,**B**) and HPrEpiC (**C**) cells. Control cells (CTRL) were not treated. Results are means ± SEM. * *p* < 0.05, and *** *p* < 0.001 vs. ctrl; ^§§^ *p* < 0.01 and ^§§§^ *p* < 0.001 vs. PIC or LPS.

In macrophages, TLR signaling promotes oxidative phosphorylation (OXPHOS) activity and the consequent increased generation of ROS [39] Due to the inhibitory activity exerted by PC and PEA on ROS production observed in this study, we wondered whether the effect could be mediated by a respiratory modulation. To test this possibility, mRNA transcription of mitochondrial respiratory complexes was evaluated in both cell lines. After Poly(I:C) incubation (Figure 5A) and LPS treatment (Figure 5B), we found an increased mRNA level of cytochrome *c* oxidase subunit 2 (COXII) and subunit 4 (COXIV), two subunits of complex IV whose transcripts are of mitochondrial (the former) and nuclear (the latter) origin (Figure 5A,B). Moreover two subunits of ATP synthase were upregulated in their mitochondrial (MT-ATP6) and nuclear (ATP5B) transcription (Figure 5A,B). Furthermore, we demonstrated that PC and PEA hampered the mitochondrial respiratory burst induced by bacterial or viral stimuli in Beas-2B (Figure 5A,B). The same results were obtained when the prostate HPrEpiC cells were treated with Poly(I:C), PC and PEA (Figure 5C). Altogether the data collected suggest that the mixture of the two substances protects against a massive mitochondrial respiratory engagement, contributing to limiting ROS production (a byproduct of mitochondrial activity) and, thus, protecting from the oxidative damage 

## 4. Discussion

In this study, we investigated the impact of PC and PEA in modulating the inflammatory response to both LPS, the major component of the outer membrane of Gram-negative bacteria, and poly(I:C), a viral mimic, in human normal bronchial and prostate epithelial cell lines. The lung and prostate epithelial tissue are often subject to acute and chronic inflammatory conditions; hence, these models are worth of investigation regarding the potential for new anti-inflammatory treatments in vitro. Considering that the inflammatory status triggered by the infectious stimulation is characterized by the reciprocal control of three main mediators (COX-2, ROS and the antioxidant glutathione), we sought to investigate the effects of PC and PEA on this pro and anti-inflammatory triad.

The novelty of this study is shown by the conclusions drawn from the following results:

1. The direct inhibitory activity exerted by PEA on COX-2.
2. The potentiation of antioxidant and anti-inflammatory activity exerted by the combination PC and PEA, which is due to a synergy in inhibiting COX-2 and consequent ROS production.
3. The induction of glutathione synthesis by the combined treatment.
4. The inhibition exerted by the two molecules on the respiratory burst triggered by bacterial or viral stimuli.

We confirmed that in vitro PC is able to inhibit human recombinant COX-2, the enzyme which mediates the bioconversion of arachidonic acid to inflammatory PGs [40], with a calculated IC50 compatible with the values described in previous studies [18], and we demonstrated, for the first time, that PEA is also a direct COX-2 inhibitor. This mechanism was not shown before, as the anti-inflammatory effect of PEA was mainly ascribed to PPARα signaling. A previous study carried out in stimulated macrophages reported that, although PGE2 levels were affected by PEA, no differences in COX-2 activity or expression were evident, suggesting the possibility of an indirect effect of PEA on the COX-2 signaling pathway [41].

Most interestingly, in our study we demonstrated a potentiation of ten times when the molecules were combined to inhibit COX-2, compared to the effect of PC alone. Moreover we found that the inhibitory concentration of the two compounds in combination is similar or even lower compared to that of specific COX-2 targeting drugs [42,43,44]. This important consideration fits well with the need to explore and evaluate alternative molecules with COX-2 inhibitory activity [18] to address two key issues: (1) nonsteroidal anti-inflammatory drugs (NSAIDs), commonly used as anti-inflammatory agents in the treatment of acute or chronic pain, simultaneously inhibit COX-2 and COX-1, the latter in turn significantly affecting the gastrointestinal and renal functions [45]; (2) the recent withdrawal from the market of some selective COX-2 inhibitors, named COXIBs. Indeed, although selective COX-2 inhibitors show superior therapeutic efficacy in the modulation of inflammatory processes, unfortunately it was discovered that their structure is behind the adverse cardiovascular side effects of selective COX-2 inhibitors [45,46]. 

In our assays on cells, the stimulation paradigm included LPS and poly(I:C), mimicking the microbial and viral infection, respectively. One of the mechanisms involved in LPS-induced inflammation and OS is the activation of the TLR4 and the translocation of NF-κB [47], which leads to an increased expression of COX-2 [48]. COX-2, the inducible isoform of cyclooxygenase and the rate-limiting enzyme in PGE2 synthesis, is rapidly expressed in response to proinflammatory molecules, and is itself a proinflammatory mediator [49]. Poly(I:C) is also able to induce a cytokine response activating a distinct IFN-regulatory factor 3 (IRF3) and NF-kB signaling during type-I IFN and TNF responses in human macrophages [38]. Both LPS and poly(I:C) trigger cytokine induction to participate in the clearance of the microbes and to defend the host [50]. 

First, our in vitro experiments verified that LPS and poly(I:C) acted through COX-2-mediated prostaglandin production and found that PGE2 secretion was blocked by PC and PEA, most potently in combination. The essay confirmed the results obtained by the analysis of IC50. 

Further experiments showed the oxidant properties of LPS and poly(I:C), and the increased production of ROS was again abated by single and combined treatment with PC and PEA. While PC is a known scavenger of ROS, a similar scavenging mechanism was not described for PEA. A previous study found that PEA downregulated the expression of TLR4, suggesting that the antioxidant effect of PEA was mediated by TLR4-dependent PPARα activation [51]. In our study we demonstrated an additional novel mechanism of PEA, the direct inhibition of COX-2, which can explain its antioxidant properties in addition to PPARα activation. Therefore, the combination PC/PEA could exert both direct and indirect effects on ROS production, the former mediated by direct neutralization of ROS, the latter mediated by inhibition of COX-2-dependent production of free radicals and consequent decrease of cytotoxicity. 

In living cells, ROS mediate intracellular and intercellular signaling, and all cells contain specific systems that guard redox homeostasis. These systems include both ROS-producing and ROS-neutralizing enzymes. It is known that the excessive production of ROS induced by infection activates NF-kB, which orchestrates the gene expression involved in the inflammatory response. Cellular redox homeostasis is largely controlled by glutathione levels, as this peptide fulfills several essential functions, among them being detoxification from free radicals and toxic oxygen radicals, thiol-disulfide exchange and storage and transfer of cysteine. Mitochondrial GSH is of paramount importance in protecting the organelle from ROS produced during coupled mitochondrial electron transport and oxidative phosphorylation [52]. For these reasons, we decided to investigate whether the protection exerted by PC and PEA against free radical overproduction was mediated by a modification of glutathione levels. Interestingly, intracellular glutathione content was greatly enhanced by cotreatment with poly(I:C), PC and PEA, and a significant increase was observed with LPS, PC and PEA. In agreement with these results, analysis of the biosynthetic pathway revealed induced expression of the key enzyme GCL. Combining the results relative to ROS production and GSH modulation, we reached some interesting conclusions. First, the combined treatment with PC and PEA both abated ROS production and induced GSH biosynthesis, leading to increased GSH availability. This observation is novel for the two molecules, but is similar to what was reported for other natural compounds such as anthocyanins and flavonoids. In fact, it has been shown that a mixture of anthocyanins has antioxidative properties resulting from radical scavenging activity, increased intracellular GSH through induction of GCL and recovery of intracellular ROS [53]. Moreover, various flavonoids and quercetin were able to increase the intracellular concentration of glutathione and regulated GCL gene expression [54]. Second, poly(I:C) is a strong pro-oxidant stimulus which triggers GSH biosynthesis as a compensatory mechanism. In our cellular model LPS does not evoke the same response on redox homeostasis, although in other studies LPS was able to induce GSH synthesis through nuclear factor-erythroid 2–related factor 2 (Nrf2) signaling [55]; probably, in Beas-2B cells the LPS-driven production and neutralization of ROS are balanced by synthesis and utilization of GSH and the variation of GSH homeostasis is not detectable. Because both the infection mimickers and PC/PEA are able to stimulate GSH production, and PC/PEA are ROS-buffering molecules which prevent GSH consumption, the cotreatment greatly enhances GSH levels. PC and PEA can support GPX activity and the consequent decrease of COX-2 by increasing GSH availability and can prevent undue responses to inflammatory stimuli. Altogether, our data demonstrate that PC and PEA can potentiate the natural intracellular antioxidant defenses and are protective against oxidant stressors such as infectious agents. 

This study also revealed the anti-inflammatory properties of combined PC and PEA. Notably, PC and PEA reduce the secretion of inflammatory cytokines induced by bacterial or viral stimuli. These effects are enhanced by the combination of PC and PEA, mostly upon the most potent stimulation with poly(I:C). The anti-inflammatory effect may be the consequence of the inhibited COX-2 activity. In conclusion, we demonstrated that the antioxidant effect of PC and PEA is mediated by COX-2 direct inhibition (greatly potentiated by the combination) which decreases ROS production. In turn, the curtailed ROS levels prevent cytokine production triggered by the inflammatory stimulation, thus explaining the anti-inflammatory mechanism of PC and PEA. Moreover, for the first time, we show a novel antioxidant and anti-inflammatory mechanism, i.e., an increase of the natural antioxidant defense molecule glutathione.

Finally, another interesting discovery ensued from the analysis of respiratory activity measured as transcription of several elements of the respiratory chain and OXPHOS. This investigation was suggested by the published characterization of a novel pathway by which macrophages generate ROS in response to bacteria by coupling TRL signaling to mitochondrial complex I via the TRAF6-ECSIT complex [39]. ROS production can lead to “ROS-induced ROS release”, a vicious circle in which ROS species increase the permeability of mitochondrial pores leading to mitochondrial dysfunction and to further ROS release [56]. A recent work investigated the mechanisms of respiratory damage triggered by ROS, and demonstrated that ROS modulate the expression and transcription of the respiratory chain and that the upregulation of complex IV due to ROS overload can be prevented by the antioxidant *N*-acetylcysteine [57]. In agreement with these studies, we detected increased transcription of respiratory elements induced by bacterial or viral stimuli and found that PC and PEA strongly abated the respiratory burst. This effect has never been described before and suggests that the mixture of the two substances protects against respiratory derangement by their antioxidant activity. 

Based on the evidence of this study, which demonstrated the ability of PC and PEA to abate the signaling of LPS and PIC, and similarly to the anti-inflammatory mechanism reported for several different molecules which control the LPS/TLR/NF-kB axis [58,59], we expect that PC and PEA activities should also be mediated by the same pathways. Our future research will involve a more detailed analysis of their signaling, with the aim of verifying and perhaps unveiling some new molecular detail.

An imbalance between the antioxidant capacity and the production of ROS leads to oxidative stress, which is associated with the pathogenesis of several human diseases [60]. Oxidant/antioxidant imbalance and the excessive production of proinflammatory cytokines are positive mediators of inflammation [61]. Systemic inflammation has emerged as an important factor for the occurrence and/or progression of inflammatory disorders [48,49]. Notably, infection has gradually been accepted as a major driver of inflammation-induced tumorigenesis [62]. For these reasons, controlling inflammation is critical in preventing various diseases, such as allergies, autoimmune and neuronal diseases, metabolic syndromes, cancer and aging processes [63,64]. Thus, the development of agents that can control systemic inflammation and/or microglial activation is suggested as an important therapeutic strategy for inflammatory diseases [65]. Based on the mounting interest in using nutraceuticals as adjuvants in clinical practice [17], our in vitro study has unveiled new mechanisms of action, and the enhanced efficacy of PC and PEA, supporting possible exploitation of this combination in human disorders. 

## 5. Conclusions

**Figure 6:**
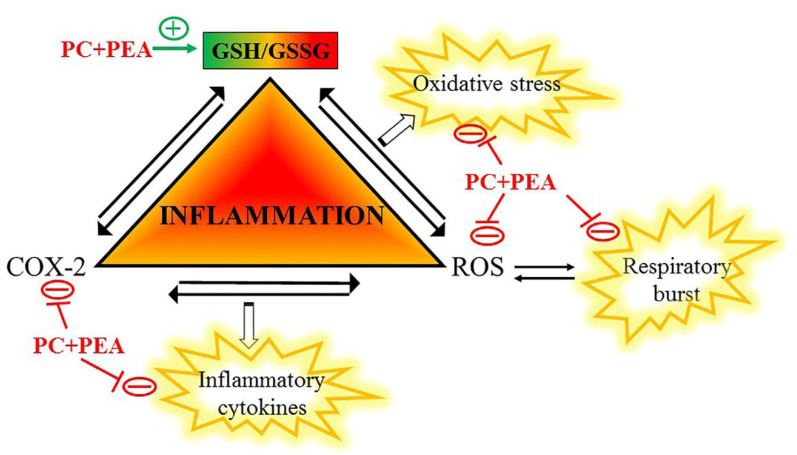
A working model of the molecular mechanisms underlying the antioxidant and anti-inflammatory effects of PC and PEA.

This in vitro analysis supports the validity of the simultaneous administration of PC and PEA in protecting against cytotoxicity and counteracting both oxidative stress and inflammation. These results suggest that the treatment with PC and PEA prepares the cell to respond more decisively to the inflammatory stimulus by increasing glutathione and, thereby, enhancing the natural antioxidant and anti-inflammatory defenses of the tissue. The beneficial effects are exerted through the following mechanisms: (1) reduced secretion of inflammatory cytokines, and (2) decreased mitochondrial respiratory burst induced by bacterial or viral stimulus, with consequent abatement of ROS levels and inhibition of the self-amplifying loop ROS/COX-2/inflammatory cytokines. Moreover, PC and PEA protect the cell from oxidative stress by affecting three main pathways: (1) inhibition of COX-2 and consequent decrease of signaling generating ROS; (2) increased synthesis of glutathione and, thereby, strengthening of the natural antioxidant defenses of the tissue, and (3) decreased infection-driven mitochondrial respiratory burst, which generates oxidative stress. A schematic representation of the influence exerted on key players of inflammation is shown in Figure 6.

## Data Availability

The data presented in this study are available within the article.

## Supplementary Materials

The following supporting information can be downloaded at https://www.mdpi.com/article/10.3390/antiox11020201/s1. Table S1: Gene accession number of amplified human genes. Figure S1: PC and PEA exert a dose-dependent and synergic inhibition of COX-2 activity.
