# Supplementary material for: Antioxidant and Anti-Inflammatory Activity of Combined Phycocyanin and Palmitoylethanolamide in Human Lung and Prostate Epithelial Cells
Source: Antioxidants (Basel). 2022 Jan 21;11(2):201. doi: 10.3390/antiox11020201 (PMC8868053; doi:10.3390/antiox11020201)
Supplement: Supplementary file 1 [file antioxidants-11-00201-s001.zip › antioxidants-1477548-supplementary.pdf]

Supplementary Materials

Table S1. Gene accession number of amplified human genes.

**IL-8**	**M28130**
IL-6	Y00081
TNF-α	X02910
cytochrome /*c*/ oxidase subunit 2	V00662
cytochrome /*c*/ oxidase subunit 4	AF017115
ATP synthase subunit beta (ATP5B)	M27132
ATP synthase F0 subunit 6 (MT-ATP6)	J01415
glutamate-cysteine ligase catalytic subunit (GCLC)	AY780794
beta 2-microglobulin (β2M)	M17987

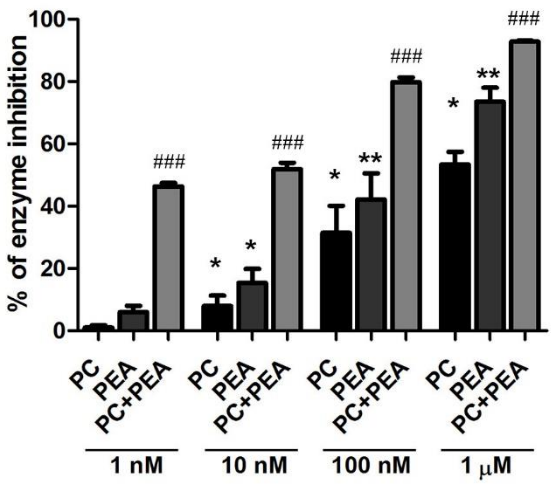

Figure S1. PC and PEA exert a dose-dependent and synergic inhibition of COX-2 activity.
